# A comprehensive overview of exosomes in ovarian cancer: emerging biomarkers and therapeutic strategies

**DOI:** 10.1186/s13048-017-0368-6

**Published:** 2017-11-03

**Authors:** Lin Cheng, Shuying Wu, Kun Zhang, Yun’an Qing, Tianmin Xu

**Affiliations:** The Second Hospital of Jilin University, Jilin Changchun, 130041 People’s Republic of China

**Keywords:** Exosomes, Ovarian cancer, Protein, RNA, DNA

## Abstract

Exosomes are nanoparticles(40-100 nm) secreted by most cells in the body, which can be isolated from several types of extracellular fluids. It has been shown that exosomes play a key role in intercellular communication and in transportation of genetic information. Emerging evidence shows that exosomes are mediators of metastasis in tumour cells, stromal cells and the extracellular matrix component through the shuttling of cargo, such as proteins, lipids, RNAs, double-stranded DNAs, non-transcribed RNAs, and microRNAs. This phenomenon has been indicated in both tumourigenesis and drug resistance. In this review, we introduce new methods of exosome extraction, focusing on the emerging role of exosomes in ovarian cancer, and discuss their potential clinical applications.

## Background

Exosomes were first used in 1981 to describe exfoliated vesicles with 5′-nucleotidase activity [1]. A few years later, Stahl’s group discovered externalized vesicles, which were thought to discard unwanted transferrin in maturing sheep reticulocytes [2]. At present, exosomes specifically refer to nanoparticles(40-100 nm) and are classified as endogenous cellular components that originate from multivesicular bodies(MVBs), which form by the inward budding and fission of late endosomes.

Little progress was made regarding exosomes until Raposo’s group [3] observed that B lymphocytes stimulate T cells proliferation by secreting exosomes containing functional MHCI, MHCII and T cells costimulatory molecules, which were reported to suppress tumour growth. Although the biological functions are not well-defined, exosomes are known to exist in almost all types of extracellular fluids (blood, urine, amniotic fluid, saliva, ascites, milk, seminal fluid and cerebrospinal fluid). They also carry many bioactive molecules, which suggests that the secretion of exosomes is a general cellular function. Therefore, exosomes play a significant role in intercellular communication by transferring both proteomic and genomic materials between cells.

Evidence shows that exosomes are released more vigorously in pathological conditions. They are released by a variety of tumour cells, and are present in large numbers in specimens from patients with different types of cancer, as well as cancer cell supernatants [4]. Exosomes derived from tumour cells were also found to express some tumour special factors which many be implicated in clinical applications for the diagnosis, prognosis and potential treatment of certain cancers [5]. For example, exosomes derived from pancreatic carcinoma which contain elevated levels of a specific proteoglycan, may serve as potential non-invasive diagnostic biomarkers to detect early stages of cancer [6].

Ovarian cancer(OvCa) is among the most common types of cancer and is the leading cause of death from gynaecological malignancies in the world [7]. More than half of OvCa patients are in an advanced stage when they see their doctors. The low survival rate and poor quality of life for patients with OvCa is in part due to the lack of early diagnostic methods and high chemoresistance rate. Therefore, it is critically emerging to to further understand the mechanisms of OvCa pathophysiology in order to uncover more precise clinical applications in the diagnosis, prognosis and treatment of the disease.

Exosome research has rapidly expanded over the last decade. For example, it has been reported that malignant ascites-derived exosomes of OvCa might augment tumour invasion [8]. We believe the exosomes has strong therapeutic potential for the diagnosis and treatment of OvCa. Therefore, it is necessary to educate clinicians and researchers in the field of Ovca in regards to exosomes. The main objective of this review is to describe recent progress in exosome research, especially therapeutically in the field of Ovca, focusing on the potential role of exosomes as novel biomarkers, as well as to introduce new methods of exosome extraction.

## Characteristics of Exosomes

**Fig. 1:**
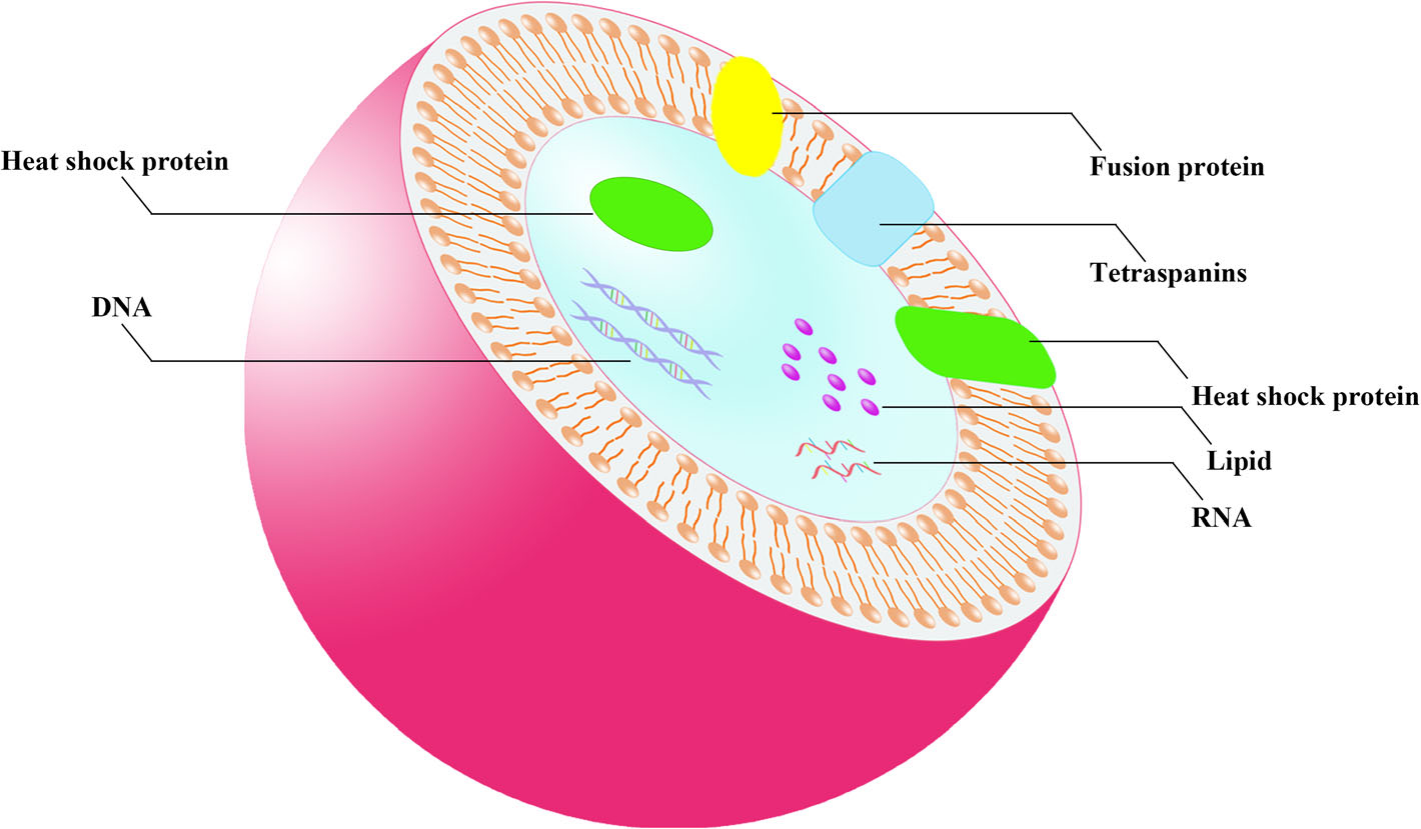
Schematic diagram of exosomes. Exosomes possess a double membrane structure-like cell membrane. They contain several types of bio-active molecules, such as proteins, lipids, RNAs, and DNAs

Exosomes typically show a “cup-shaped” or “saucer-like” morphology when analysed by electron microscopy [9]. In addition, they can float at 1.1–1.18 g/ml in sucrose density gradient [10]. The surface of exosomes is characterized by the presence of multiple families of proteins, such as tetraspanins (CD63, CD81, CD9), heat shock proteins (Hsc70), lysosomal proteins (Lamp2b) and fusion proteins (CD9, flotillin, Annexin) [11]. The tetraspanins have been used as exosome markers to distinguish them from microvesicles, apoptotic bodies and other vesicles. However, a precise exosome-specific biomarker has not yet been discovered [12]. Exosomes also contain several types of bio-active molecules, such as proteins, lipids, mRNAs, microRNAs (miRNAs), long non-coding RNAs (lncRNAs), genomic DNA, cDNA, and mitochondrial DNA (mtDNA) [13–19]. For a more extensive discussion on the molecular cargos of exosomes, the reader should refer to ExoCarta (http://www.exocarta.org), an exosome database, providing the exosome contents identified in multiple organisms [13, 20]. The current version contains 41,860 proteins entries, 4946 RNA entries and 1116 lipid entries from 286 studies (Fig. 1).

## Ovarian cancer-derived Exosomal cargos and their role as potential biomarkers

Compared with conventional cancer biomarkers, exosomal cargos have characteristic features that differentiate them from non-cancer exosomes. They have similar, and sometimes higher, that can be specificity and sensitivity attributed to their origin. They have excellent stability, and are detected in all body fluids, particularly in peripheral blood. These features make exosomes a potentially ideal biomarker of cancer [21].

Many different molecular cargos identified in OvCa-derived exosomes have drawn much attention due to their large potential in (i) early diagnosis, (ii) prognosis, (iii) drug resistance, and (iv) targeted therapy of OvCa.

### Proteins

To date, over 2000 species of protein have been identified from OvCa-derived exosomes according to ExoCarta. Their involvement in tumour progression and metastasis has been reported in many of these identified proteins, including membrane proteins (Alix, TSG 101), and tetraspanins (CD24, CD44, CD63, CD37, CD53, CD81), as well as enzymes (phosphate isomerase, peroxiredoxin, gelatinolytic enzymes, aldehyde reductase). Moreover, a study by Liang et al. illustrated that OvCa-derived exosome proteins were highly enriched in signal pathways associated with carcinogenesis. They found that a subset of proteins overexpressed in ovarian cancer tissue were present in the exosomal protein list, including epithelial cell surface antigen (EpCAM), proliferation cell nuclear antigen (PCNA), tubulin beta-3 chain (TUBB3), epidermal growth factor receptor (EGFR), apolipoprotein E (APOE), claudin 3 (CLDN3), fatty acid synthase (FASN), ERBB2, and L1CAM (CD171). These protein may also be a source of diagnostic markers and targets for therapeutic methods for Ovca [22]. Among them, EpCAM has been extensively studied and, is used as a biomarker or prognostic factor of many cancers. For example, Huang et al. demonstrated an intricate relationship between EpCAM-regulated transcription and altered biophysical properties of cells that promote EMT in advanced endometrial cancer [23]. However, there are drawbacks to the use of EpCAM as an OVCA biomarker. For example, Shen’s group compared the characteristics of exosomes derived from human ovarian epithelial cells (HOSEPiC) and three OvCa cell lines (OVCAR3, IGROV1, and ES-2), and found that the labeled rates by anti-EpCAM antibodies were 16.4, 23.7, 15.7, and 18.5%, respectively suggesting that EpCAM may not be an appropriate marker for detecting early stages of OvCa [24]. The negative outcome may have been due to the fact that EpCAM can be cleaved from exosomes via serum metalloproteinase [25]. While some articles suggest an outlet for combining EpCAM with CD24 to detect Ovca-derived circulation exosomes, large scale clinical trials is needed to verify this hypothesis. In terms of the protein CLDN3, Morin’s group found that CLDN3 was not likely to represent a useful biomarker compared to CLDN4, which had a sensitivity of 51% (32/63) and specificity of 98% (49/50) for the detection of OvCa [26].

Exosomal proteins also have the potential to serve as tumour staging and prognostic markers for response to treatment of ovarian cancer. Marta Szajnik et al. found that plasma from OvCa patients contained higher levels of exosomal proteins compared to plasma from patients with benign tumors or NCs (healthy controls). Furthermore, the exosomal protein content was significantly higher in advanced stages than that of early stages. In further studies, they found that TGF-β1 and MAGE3/6 could distinguish OvCa patients from those with benign tumours and NC. Moreover, the exosomal protein levels variably changed and were correlated with chemotherapy efficacy [27].

Early detection of the resistance to platinum-based therapy is critical for improving the treatment of Ovca. Increased expression of annexin A3 is a mechanism for platinum resistance in ovarian cancer, which is associated with exocytosis and the release of exosomes [28]. For therapy potential, the exosomal ADAM15 ectodomain effectively inhibits cancer progression by blocking the integrin-mediated MEK/ERK signalling pathway, providing insight into the functional significance of exosomes that generate tumor-inhibitory factors [29].

### MicroRNAs

MicroRNAs (miRNA), small (22–25 nucleotides in length) noncoding RNAs, inhibit gene expression post-transcriptionally by binding to their 3′untranslated region, leading to the suppression of protein expression or cleavage [30, 31]. Several studies have accounted for theirThey are involvinvolvemented in carcinogenesis, the cell cycle, apoptosis, proliferation, invasion, metastasis, and chemoresistance [32, 33].

For instance, the levels of exosomal miR-200b and miR-200c were found to be higher in patients with FIGO stage III–IV, including lymph node metastasis compared to patients with FIGO stages I–II, suggesting that these microRNAs may be involved in tumour progression [34]. Exosomal miR-21-3p can also contribute to cisplatin resistance by potentially targeting the NAV3 gene. MiR-21 in exosomes and tissue lysates isolated from cancer-associated adipocytes (CAAs) and fibroblasts (CAFs) are transferred from CAAs or CAFs to cancer cells, where they suppress ovarian cancer apoptosis and confer chemoresistance by binding to the direct novel target, APAF1 [35].

Furthermore, miR-222-3p is enriched in OvCa-derived exosomes, and can be transferred to macrophages to induce a tumour-associated macrophage (TAM)-like phenotype with SOCS3/STAT3 pathway involvement, potentially facilitating the progression of cancer [36]. From a prognostic standpoint, the exosomal miRNAs miR-21, miR-103, miR-141, miR-203, miR-205, miR-214, miR-373, and miR-200a-c are associated with a poorer prognosis. For more information on exosomal miRNAs in OvCa, readers may refer to Table 1.

**Table 1:** MicroRNA content of ovarian cancer-derived exosomes

microRNAs	Origin	Possible biological significance
miR-21,miR-103,miR-141,miR-203,miR-205,miR-214,miR-373,miR-200a&b&c [34, 37]	Serum	Diagnostic and poor prognosis
miR-21-3p [38],	A2780	Drug resistance
miR-30a-5p [39]	urine	Diagnostic
LIN28A [40]	IGROV1	Invasion and Migration
miR-222-3p [36], Let-7, miR-200 [41]	Skov-3	Diagnostic and Invasion
miR- 21, miR- 23b, miR-29a, miR-99a, miR-125b, miR-200c, miR-320a and miR-484 [42]	ES2 Effusion	Poor survival and Clinicopathologic Parameters
miR21 [35]	OVCA432 SKOV3	Drug resistance

Although exosomes have been proposed as vehicles for microRNA (miRNA)-based intercellular communication and sources of miRNA biomarkers, and providers of information on aberrant signalling pathways, some disputes remain. By studying cancer-associated extracellular miRNAs in patient blood samples, John R. Chevillet et al. found that exosome fractions contained a small minority of the miRNA content of plasma. Their data suggests that most individual exosomes in standard preparations do not carry biologically significant numbers of miRNAs and are, therefore, unlikely to be functional independently vehicles for miRNA-based communication [43].

### Other cargos

With the exception of proteins and miRNAs, several bio-active molecules such as phosphatidyl-serine (PS), DNAs, glycans, and glycoprotein [44], have been reported to play an important role in exosome internalization, signal recognition, and novel vaccines. Early evidence suggests that uptake of OvCa derived-exosomes by NK cells requires PS at the exosomal surface, but the presence of PS is not sufficient [8]. The latest study by Jayanthi Lea et al. also provides proof of concept data supporting the high diagnostic power of PS detection in the blood of women with suspected ovarian malignancies [45]. Exosomes were found to contain complex glycans of the di-, tri-, and tetraantennary type with or without proximal fucose, as well as high levels of mannose glycans. The sialoglycoprotein galectin-3-binding protein (LGALS3BP) was found to be strongly enriched in exosomes, making it a potential marker for exosomes [46]. Scientist have highlighted the translational value of exosomal DNA (exoDNA) in tumour-derived exosomes due to its potential usefulness as a circulating biomarker for the early detection of cancer and metastasis. ExoDNA represents the entire genome and reflects the mutational status of parental tumour cells providing the following advantages: (i) its protection, and, thus inherent stability within exosomes [47], (ii) enriched tumour-derived exosomes found in complex plasma samples [16, 48].

## Roles in ovarian cancer progression and metastasis

Unlike most solid tumours, OvCa rarely disseminates through vasculature, but has a high propensity to metastasize within the peritoneum. This allows tumour cells to directly encounter human peritoneal mesothelial cells (HPMC) in the initial step of metastasis. In this seemingly “Pandora’s box”, exosomes appear to be a new and powerful signal mediator by cleaning the mesothelial barrier for improved cancer cell invasion [49, 50]. For instance, OvCa derived exosomes contain gelatinolytic enzymes, the L1 adhesion molecule (CD171) and other cell adhesion molecules [51]. Koji Nakamura et al. observed that HPMCs underwent a change in cellular morphology to a mesenchymal, spindle phenotype when they internalized OvCa-derived exosomes [50]. The formation of malignant ascites is often observed in advanced OvCa patients. Several studies revealed that malignant ascites-derived exosomes contain multiple cargos, such as L1CAM, CD24, ADAM10, Claudin-4 and EMMPRIN which play a critical role in tumour progression [8, 52].

In recent years, the tumour microenvironment, including stromal cells, endothelial cells, infiltrating immune cells, and the extracellular matrix [53] has been recognized as having a critical role in OvCa metastasis. Exosomes appear to be a novel and significant signalling factor in the tumour microenvironment. In OvCa, high LIN28A expressing OvCa cells derived from exosomes induce EMT-related (epithelial to mesenchymale transition) gene expression, invasion and migration when taken up by non-metastatic target cells [40]. In regards to immune cells, malignant ascites-derived exosomes may induce apoptosis of the precursors of DCs (peripheral blood lymphocytes) and PBMCs (dendritic cells) [54], and may contain immunosuppressive factors, such as TGF-β1 and IL-10, which indicate that exosomes may be involved in the support of immune evasion in OvCa [55]. Further study by Alireza Labani-Motlagh et al. revealed two cytotoxic pathways of importance for anticancer immunity: the NKG2D receptor-ligand pathway and the DNAM-1-PVR/nectin-2 pathway [56]. In addition, exosomes may induce adipose-derived mesenchymal stem cells (ADSCs) to acquire a tumour-supporting myofibroblast phenotype and functionality. They also may activate macrophages to possess a tumour-associated macrophage (TAM)-like phenotype, which could facilitate the progression of cancer [4, 36]. More recently, it was reported that CAA (cancer-associated adipocytes) and CAF (cancer-associated fibroblasts) derived exosomes could increase chemoresistance by transporting microRNAs to surrounding cancer cells [35]. Miharu Kobayashi et al. found that significantly less let-7 family miRNA was expressed in high invasive cells (SKOV-3), but was highly expressed in exosomes. Within a tumour, high invasive tumour cell derived exosomes signal low invasive tumor cells and low invasive tumour cell derived-exosomes can signal high invasive tumour cells to increase invasion of recipient cells [41]. This suggests that the exosomes plays an important role in the tumour microenvironment and OvCa metastasis.

## Roles in ovarian cancer therapy

### Exosome-related immunotherapy

Exosomes are known to contribute to immunosuppression and tumour immunity escapes [54–56]. A previous study indicated that depletion of peritoneal macrophages by clodronate could reduce ovarian tumour progression in vivo [57]. Inspired by the idea that macrophages may serve as attractive targets for therapeutic intervention, Yuan Hu et al. observed that exosomes derived from TWEAK-stimulated macrophages (TMs) could be internalized by OvCa cells and inhibit metastasis through the shuttling of miR-7, subsequently leading to the downregulation of EGFR/AKT/ERK1/2 signalling pathways [58]. Some believe that exosomes contain cell surface cancer antigens, suggesting a potential for therapeutic approaches in cancer vaccination [44]. More recently, a plateau phenomenon has been reported, suggesting that release of exosomes is regulated by a feedback mechanism regardless of tissue specificity. This phenomenon indicates that this feedback mechanism can be inhibited by various exosomes, providing a potential therapeutic approach to control the release of exosomes from OvCa cells [59].

### Exosome-facilitated drug delivery

Exosomes contain the following advantages when it comes to their potential as cell-based therapeutic products: (i) Therapeutic biological materials, such as chemotherapeutics [60], miRNAs [61] and siRNAs [62, 63], could be loaded into exosomes. For example, a recent study showed that human adipose mesenchymal stem cell-derived exosomal-miRNAs are critical factors for inducing antiproliferation signalling to A2780 and SKOV3 ovarian cancer cells [61]; (ii) Exosomes are taken up by acceptor cells, through which cellular processes can be altered [64]; (iii) Exosomes possess a high histocompatibility and do not induce immunological rejection. To achieve cell-specific targeting drug delivery, several studies have tested donor cells, loading methods and theraputic cargos of exosomes (Table 2).

**Table 2:** Types of donor cells, disease model, therapeutic cargos and loading methods

Donor cell	Disease model	Cargo	Loading method
hAMSCs [61]	Ovarian cancer	miR	–
HeyA8, SKOV3-ip1, A2780 [65]	Ovarian cancer	miR-6126	Transfection
MDA-MB-231,STOSE, CD63-GFP [60]	Breast and ovarian cancer	doxorubicin	Electroporation
BM-MSCs [66]	Glioblastoma Multiforme	Cy5-Anti-miR-9	Transfection
BM-MSCs, SR4987 [67]	human pancreatic adenocarcinoma	Paclitaxel	Incubation
BM-MSCs [68]	Osteosarcoma	miR-143	Transfection
BM-MSCs, Placenta and cord derived MSCs [69]	Glioma	miR-124, miR-145	Transfection
Adipose tissue -derived MSC [70]	Hepatocellular Carcinoma	miR-122	Transfection

## Methods of exosome extraction

**Fig. 2:**
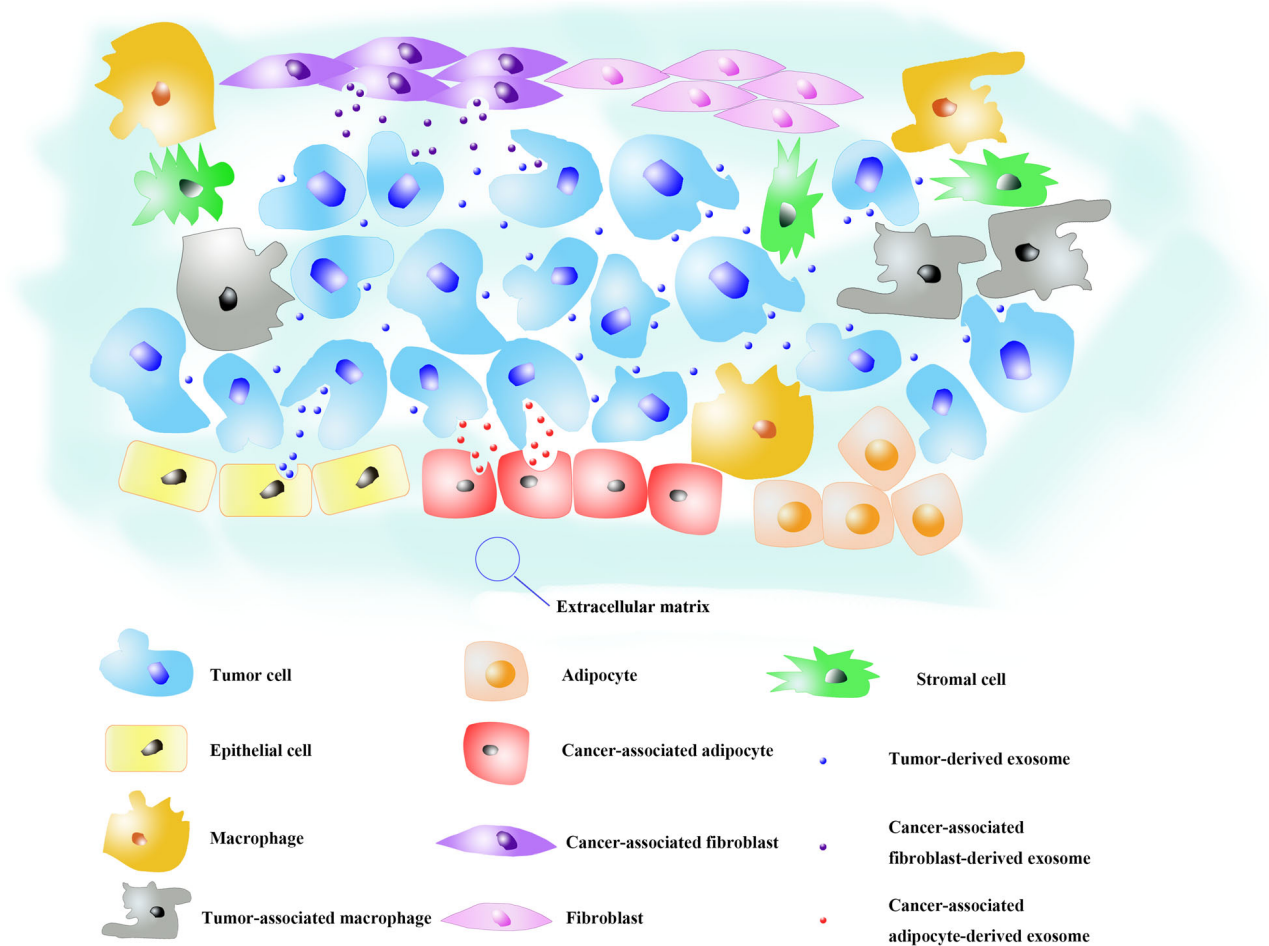
Exosomes in tumour microenvironment. As a significant signalling delivery that shuttles between stromal cells, endothelial cells, and infiltrating immune cells, exosomes promote ovarian cancer progression and metastasis by inducing normal cells (adipocytes, macrophage, fibroblasts) to acquire a tumour-supporting phenotype and functionality

There are various validated methods for exosome extraction, including ultracentrifugation, ultrafiltration, chromatography, polymer-based precipitation and affinity capture on antibody-coupled magnetic beads, which have been described exhaustively by Antes’s group [71]. In this section, we introduce new methods of exosome extraction (Fig. 2).

### Microfluidic techniques

Microfluidic technology has been previously shown to have unique advantages in genomics, proteomic analysis and quantitative biology. This method can also be used to separate exosomes from several body fluids. Mohsen Akbari [72] categorized the microfluidic systems developed for the detection/characterization exosomes into six groups: (i) electrochemical [73, 74], (ii)electrostatic potential [75], (iii) mechanical [76], (iv) electromechanical [77], (v) optical, and (vi) non-optical based.

#### Silica nanostructured platform for affinity capture

**Fig. 3:**
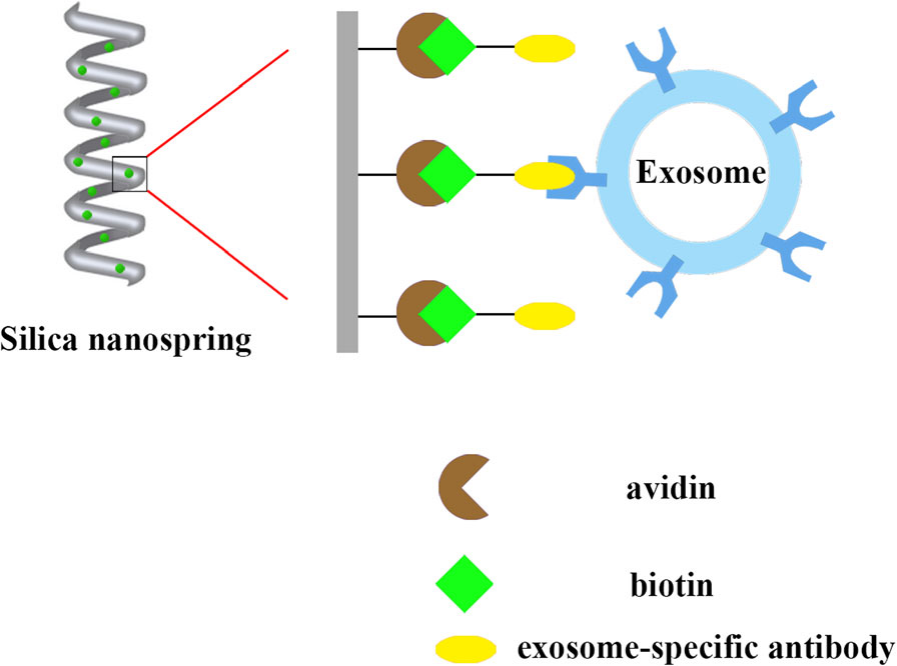
Schematic for silica nanostructured platform

Similar to other affinity capture methods, the silica nanotechnology platform selectively captures specific exosomes based upon certain surface markers. This approach employs silica nanosprings, made of silica glass with unique physical and chemical properties which consist of a wide gravimetric surface area tethered to Si wafer substrates, creating a broad space for affinity capture [78], as shown in Fig. 3 of how this works. Norton’s group also reported that the biotin binding capacity for the avidin-nanosprings was 4-fold greater than commercially available streptavidin-coated silica beads under identical conditions [78]. This method overcomes the shortcomings of conventional immuno-affinity purification, and makes large scale EV purification possible.

#### Aptasensor based on DNA-capped single-walled carbon Nanotube

Recently, Chinese scientists demonstrated a visible and simple method for the detection of exosomes by integrating DNA-capped single-walled carbon nanotubes [79]. This new method consists of two main elements: The first is s-SWCNTs, a tubular nanomaterial rich in carboxyl groups and water solubility. The other is aptamers, specific to CD63 and absorbed onto the surface of s-SWCNTs, which can efficiently catalyze H2O2-mediated oxidation of 3, 3′, 5, 5′-tetramethylbenzidine (TMB) and lead to a change from colourless to blue in solution. Therefore, the colour of the solution represents the quantity of exosomes and can be observed by the naked eye or monitored by spectrometry. Moreover, this proposed colorimetric aptasensor is universally applicable for the detection of other targets by simply changing the aptamer.

To date, microfluidic techniques show higher recovery and purity of exosomes compared to conventional methods. However, improving the throughput of on-chip isolation technologies while retaining their high particle sorting sensitivity remains an ongoing challenge in the field.

### Thermo-acoustofluidic separation

Thermo-acoustophoresis adds a temperature dimension to conventional methods, integrating a concurrent application of piezoelectric and thermoelectric effects on a single-stream flow in a microchannel to help with the separation and identification of extracellular vesicles, such as exosomes and microvesicles. As vesicles in a predetermined temperature pass through the ultrasonic radiation field, they possess a stiffness-dependent force, leading to their migration towards either the node or the anti-nodes, which is determined by the acoustic contrast factor(Φ). For a system formed by two different vesicles with distinct membrane stiffness values, the change in Φ brings out a temperature “window” in which opposite Φ signs exist. As ultrasonic standing waves are able to accommodate subtle differences, if the preinstall temperature is set to that window, vesicles are then separated at efficiencies exceeding 95% [80].

### Lipid-based nanoprobes

A lipid nanoprobe (LNP) system for the rapid isolation of extracellular vesicles (EVs) including exosomes has been reported recently. The approach includes a labelled lipid bilayer with biotin-tagged 1, and 2-distearoylsnglycero-3-phosphethanolamine-poly (ethylene glycol) (DSPE–PEG). The labelled EVs are collected by NeutrAvidin (NA)-coated magnetic sub-micrometre particles (MMPs), which shortens the isolation procedure from hours to 15 min and does not require large and expensive equipment [81]. This method is also highly flexible and can be adopted for analyses of various downstream bioactive substances, such as DNA, RNA and proteins.

## Conclusion and future perspective

Exosomes play a crucial role in multiple pathophysiological procedures, such as inflammatory response, immunoregulation, tumorgenesis, tumor invasion and metastasis. Many new findings and new hypotheses suggest the need for further research, as exosomes have great diagnostics and therapeutic potential, yet many questions remain. New methods have been explored to isolate exosomes efficiently and purely, and future studies will need to make comprehensive comparisons of these methods. Multi-Omics, such as the high-throughput expression analysis technique, will reveal critical molecules and mechanisms for the packaging of cargos.

Exosomes have shown immense potential in the early diagnosis, drug selection, prognostic evaluation, and target therapies involved in OvCa. Nonetheless, limitations of reported studies include cell lines and a sample size. Therefore, there is a critical need for multiple, large-scale clinical studies regarding the involvement of exosomes in OvCa.

## Abbreviations

ADSC: Adipose-derived mesenchymal stem cells
CAAs: Cancer-associated adipocytes
CAFs: Cancer-associated fibroblasts
EMT: Epithelial to mesenchymale transition
EVs: Extracellular vesicles
exoDNA: Exosomal DNA
HPMC: Human peritoneal mesothelial cells
lncRNAs: Long non-coding RNAs
LNP: Lipid nanoprobe
miRNAs: microRNAs
MVBs: Multivesicular bodies
OvCa: Ovarian cancer
PS: Phosphatidyl-serine
TAM: Tumor-associated macrophage
